# Editorial: RNA at a breaking point? Cytoplasmic cleavage and other post-transcriptional RNA processing in neurodevelopment and disease

**DOI:** 10.3389/fnmol.2023.1214853

**Published:** 2023-05-30

**Authors:** Monika Piwecka, Raphaelle Luisier, Catia Andreassi

**Affiliations:** ^1^Department of Non-coding RNAs, Institute of Bioorganic Chemistry, Polish Academy of Sciences, Poznań, Poland; ^2^Genomics and Health Informatics Group, Idiap Research Institute, Martigny, Switzerland; ^3^SIB Swiss Institute of Bioinformatics, Lausanne, Switzerland; ^4^UCL Laboratory for Molecular Cell Biology, University College London, London, United Kingdom

**Keywords:** non-coding RNA, RNA modification, nerve injury, local mRNA translation, miRNA, lncRNA, circRNA

The nervous system is a complex web of thousands of cell types that require appropriate integration of developmental and mature functions to harmonically respond to both internal and external stimuli. Achieving this feat requires precise coordination of gene expression programs, with alterations in mRNA levels traditionally garnering the most attention. However, recent research has shown that non-coding RNAs, which regulate cellular functions, also play a critical role in the nervous system's function and development. Regulatory RNAs, that include but are not limited to, long non-coding RNAs (lncRNA), microRNAs (miRNA) and circular RNAs (circRNAs), play crucial roles in various biological processes ranging from cell survival to specialized functions like target innervation. Such RNA species are often subjected to modifications, post-transcriptional processing, and are localized to different subcellular compartments, all of which contribute to their biological activity and specificity (Sambandan, 2017; Rajgor, 2020; Keihani et al., 2021; Samaddar and Banerjee, 2021; Zajaczkowski and Bredy, 2021). Lastly, mRNAs or their non-coding portions such as untranslated regions (UTRs) and introns can be bestowed with alternative functions independent of their coding potential and have been shown to modulate protein-protein interactions, intracellular signaling, subcellular localization and time-dependent expression (Nam et al., 2016; Andreassi et al., 2018; Huang, 2021).

The main aim of this Research Topic is to exemplify and review novel and non-canonical functions of coding and non-coding RNAs that may contribute to brain development and functioning of neural cells in homeostasis and disease. Additionally, the Research Topic covers a few distinct aspects of RNA-mediated processes that diversify gene regulatory pathways in neural cells.

This Research Topic includes two research articles and two reviews on miRNA-mediated regulatory pathways. Han et al. investigates the alteration in the expression pattern of exosome-contained miRNAs (exo-miRNAs) isolated from serum of patients suffering from vascular dementia (VaD). The level of miR-154-5p is significantly increased in the serum exosomes of VaD patients compared to healthy individuals. Using a rat model of VaD, the authors also show that the levels of oxidative stress and inflammation are significantly elevated in VaD and that in these animals miR-154-5p is upregulated in the hippocampus, cortex, and bone marrow-endothelial progenitor cells. A study by Griffiths et al. focuses on miR-200c and its known target Sirt1 mRNA. SIRT1 protein plays a central role in maintaining mitochondrial bioenergetics through the regulation of the mitochondrial fission/fusion balance, and ROS production. The authors aim to disentangle miR-200c-Sirt1 contribution to mitochondria imbalance in astrocytes and neurons during ischemic brain injury. NF-kB complex is a known driver and regulator of miRNAs, and both excessive NF-kB and upregulated miRNAs orchestrate a pathogenic gene expression program underlying Alzheimer's disease (AD) onset, and propagation and severity of the disease. The opinion article by Lukiw comprehensively reviews current insights into the pro-inflammatory effects of miRNAs and further explores the potential mechanisms by which NF-kB-regulated miRNA-mediated signaling could be leveraged to develop novel therapeutic interventions for the more effective management of AD and other age-related neurodegenerative disorders. Finally, a mini-review by Wang and Liang summarizes state-of-the-art knowledge on miRNAs involved in T lymphocyte differentiation, activation, and functioning in the context of Multiple Sclerosis (MS). The paper evaluates differentially expressed miRNA as putative diagnostic biomarkers and includes considerations about T lymphocyte-specific miRNAs as therapeutic targets for MS treatment. Two other mini-reviews within our Research Topic provide a summary of the current literature in regard to the role played by lncRNAs in the control of oxidative stress in CNS disorders. Xu and Zhang summarize observations on lncRNAs and the mechanisms they use to regulate oxidative stress, for example by interacting with miRNAs, and the implications of these interactions for CNS disorders, such as AD, Parkinson's disease, spinal cord injury and acute ischemic stroke. Wang J. et al. instead focus on one particular lncRNA called *HOTAIR*. This comprehensive overview of the regulatory functions of *HOTAIR* highlights its contribution to the pathogenesis of ischemic stroke, neurodegenerative disorders, and traumatic brain injury. The authors conclude that *HOTAIR* holds promise to be applied as a diagnostic biomarker of CNS disorders.

Another salient theme explored in this Research Topic is the interrelation between RNA biology and nerve injury and regeneration. Cao et al. report on differentially expressed circRNAs in dorsal root ganglion neurons upon central and peripheral axon injuries in rats. Tian et al. review existing studies showing evidence for altered expression of N6-methyladenosine (m6A) RNA modifiers, and functions of related modified RNAs in CNS injuries, eventually proposing potential directions in m6A research in the context of brain injuries. Another review authored by Zhang et al. delves into the epigenetic mechanisms by which non-coding RNAs and m6A methylation modulate nerve injury-induced neuropathic pain, offering valuable insights into the potential functions and recent advances in this field. The research article by Chernov and Shubayev reveals sex-specific and sexually dimorphic regulation of neurotrophic and immune genes and mRNA axonal transport, as well as differential patterns of CNS-specific microRNA precursors and specific small nucleolar RNAs in response to sciatic nerve axotomy. Regulated neuronal mRNA transport and related local translation are comprehensively reviewed by Triantopoulou and Vidaki. This extensive article elaborates on local mRNA translation in axon outgrowth and guidance, in synapse formation and plasticity, in axon regeneration, and links mRNA transport and local RNA translation to the cytoskeleton (Triantopoulou and Vidaki). Cytoskeletal dynamics and cytoskeleton defects have been also associated and described in the context of the pathogenesis of neurodevelopmental and neurodegenerative diseases. Coordination between local translation and cytoskeletal remodeling is illustrated with multiple examples. Overall, this review article is not only broad but also brings into focus fascinating biology at the interface between the cytoskeleton, RNA-binding proteins (RBPs) and RNA in neuronal cells. More about the fascinating world of RBPs and how their localization can be altered by intrinsic and extrinsic stimuli can be found in this Research Topic. In particular, Nogami et al. describe how FUS mislocalization to cytoplasmic stress granules might result from DNA damage induction, while Wang Y. et al. investigate the alteration in intercellular communications at single-cell level during the early stages of diabetic retinopathy, identifying a new subgroup of Mueller cells and pointing to their potential role in the pathogenesis of this disease.

Our Research Topic also delves into the intricacies of transcription-associated processes that diversify gene regulatory pathways in neural cells. Marshall et al. report on the role of intron-related variable number tandem repeats in promoting disease-related gene expression diversity. Sirp et al. investigate the functional mRNA isoforms of the transcription factor 4-encoding gene (TCF4), which has been associated with several neurocognitive disorders, shedding light on the potential implications of TCF4 in a number of neurocognitive disorders. Wu et al. describe the establishment of a zebrafish model of autism, where the integration of RNA-sequencing data with Gene ontology (GO), Kyoto Encyclopedia of Genes and Genomes (KEGG) and protein–protein interaction (PPI) network analysis points them to the identification of genes that can impact the social behavior deficits observed in the affected zebrafish.

The field of RNA regulation in neurons and glial cells has been flourishing in the past two decades with multiple new discoveries and concepts. Last year (2022), the first edition of NeuroRNA conference “RNA Regulation in Brain Function and Disease” was organized to discuss state-of-the-art research at the interface of RNA biology and neuroscience. A review article by Piwecka et al. summarizes the research insights into the CNS and its dysfunctions from the systems biology perspective to finer molecular and cellular scales that were presented over three days of the conference.

We enjoyed the novel insights and summaries presented in original and review articles within this Research Topic. We hope that the content of this Research Topic will be valuable for the community of researchers that are fascinated with RNA biology and RNA-mediated gene expression regulation in the nervous system, its development, health and disease.

## Author contributions

All authors listed have made a substantial, direct, and intellectual contribution to the work and approved it for publication.
